# Simulation and estimation of gene number in a biological pathway using almost complete saturation mutagenesis screening of haploid mouse cells

**DOI:** 10.1186/1471-2164-15-1016

**Published:** 2014-11-24

**Authors:** Masahiro Tokunaga, Chikara Kokubu, Yusuke Maeda, Jun Sese, Kyoji Horie, Nakaba Sugimoto, Taroh Kinoshita, Kosuke Yusa, Junji Takeda

**Affiliations:** Department of Social and Environmental Medicine, Graduate School of Medicine, Osaka University, 2-2 Yamada-oka, Suita, Osaka, 565-0871 Japan; Department of Immunoregulation, Research Institute for Microbial Diseases, Osaka University, 3-1 Yamada-oka, Suita, Osaka, 565-0871 Japan; Laboratory of Immunoglycobiology, WPI Immunology Frontier Research Center, Osaka University, 3-1 Yamada-oka, Suita, Osaka, 565-0871 Japan; Department of Computer Science, Tokyo Institute of Technology, 2-12-1-W8-60 Oookayama, Meguro-ku, Tokyo, 152-8550 Japan; Department of Infectious Disease Control, Graduate School of Medicine, Osaka University, 2-2 Yamada-oka, Suita, Osaka, 565-0871 Japan; Stem Cell Genetics, Wellcome Trust Sanger Institute, Wellcome Trust Genome Campus, Hinxton, Cambridge CB10 1SA UK; Department of Physiology II, Nara Medical University, 840 Shijo-cho, Kashihara, Nara, 634-8521 Japan

**Keywords:** Computer simulation, GPI-anchor, Haploid mouse embryonic stem cell, N-ethyl-N-nitrosourea, Saturation mutagenesis, Whole-exome sequencing

## Abstract

**Background:**

Genome-wide saturation mutagenesis and subsequent phenotype-driven screening has been central to a comprehensive understanding of complex biological processes in classical model organisms such as flies, nematodes, and plants. The degree of “saturation” (i.e., the fraction of possible target genes identified) has been shown to be a critical parameter in determining all relevant genes involved in a biological function, without prior knowledge of their products. In mammalian model systems, however, the relatively large scale and labor intensity of experiments have hampered the achievement of actual saturation mutagenesis, especially for recessive traits that require biallelic mutations to manifest detectable phenotypes.

**Results:**

By exploiting the recently established haploid mouse embryonic stem cells (ESCs), we present an implementation of almost complete saturation mutagenesis in a mammalian system. The haploid ESCs were mutagenized with the chemical mutagen N-ethyl-N-nitrosourea (ENU) and processed for the screening of mutants defective in various steps of the glycosylphosphatidylinositol-anchor biosynthetic pathway. The resulting 114 independent mutant clones were characterized by a functional complementation assay, and were shown to be defective in any of 20 genes among all 22 known genes essential for this well-characterized pathway. Ten mutants were further validated by whole-exome sequencing. The predominant generation of single-nucleotide substitutions by ENU resulted in a gene mutation rate proportional to the length of the coding sequence, which facilitated the experimental design of saturation mutagenesis screening with the aid of computational simulation.

**Conclusions:**

Our study enables mammalian saturation mutagenesis to become a realistic proposition. Computational simulation, combined with a pilot mutagenesis experiment, could serve as a tool for the estimation of the number of genes essential for biological processes such as drug target pathways when a positive selection of mutants is available.

**Electronic supplementary material:**

The online version of this article (doi:10.1186/1471-2164-15-1016) contains supplementary material, which is available to authorized users.

## Background

Genome-wide mutagenesis and subsequent phenotype-driven screening has been pivotal to a complete understanding of how complex biological processes operate in classical model organisms including flies, nematodes, and plants [1, 2]. The level of “saturation” in mutagenesis (i.e., the fraction of possible target genes identified) has been shown to be a critical parameter for this approach to determine all relevant genes involved in a biological function, without prior knowledge of the gene products [1–3]. In mammalian model systems, much effort has been expended to saturate, i.e., to disclose all the genes involved in some specific biological pathways. However, the relatively large scale and labor intensity of experiments have hampered the achievement of actual saturation mutagenesis, especially for recessive traits that require biallelic mutations to manifest detectable phenotypes [3–6]. To overcome these drawbacks, the haploid mouse embryonic stem cell (ESC) system, in which a single-hit mutation can directly lead to phenotypic changes without being compensated by the second copy of the gene, has been recently developed [7–10], and reviewed in [11].

Here, to address the issues of mammalian saturation mutagenesis, we mutagenized the haploid mouse ESCs with the chemical mutagen N-ethyl-N-nitrosourea (ENU) and subjected them to a phenotypic screening of mutants defective in various steps of the glycosylphosphatidylinositol-anchor (GPI-anchor) biosynthetic pathway.

## Results

### The GPI-anchor biosynthetic pathway as a model target of screening

**Figure 1:**
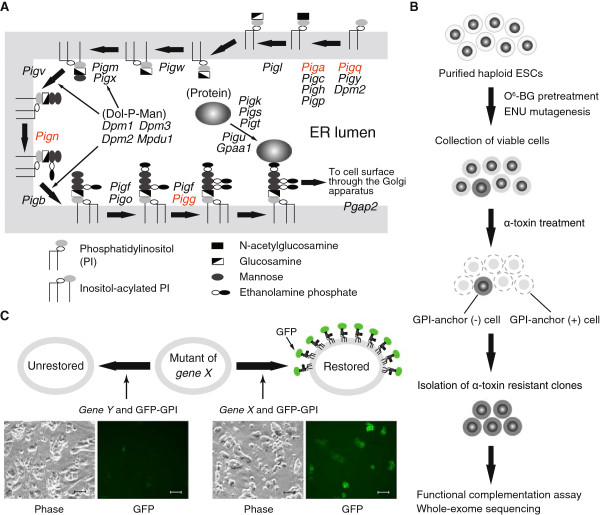
**Experimental design. (A)** Biosynthetic pathway of GPI-anchored proteins. GPI-anchor precursors are synthesized and attached to proteins in the endoplasmic reticulum (ER). The resulting GPI-anchored proteins are expressed at the cell surface. Out of 26 genes involved in this pathway, four (shown in red) were excluded from our screening (see text). Dol-P-Man, dolichol-phosphate-mannose. **(B)** Schematic screening procedure. **(C)** Functional complementation assay. Upper, principle of the assay. Lower, representative culture fields of *Pigl* ^*m*^ mutant ESCs unrestored (left) and restored (right) by plasmid transfection (scale bars, 50 μm). For clarity, original images shown in Additional file 1: Figure S1C were trimmed.

We chose the GPI-anchor biosynthetic pathway as a model target of phenotype-driven screening for a number of reasons. The GPI-anchor is a glycolipid that tethers many proteins to the plasma membrane of eukaryotic cells, forming a diverse family of molecules including hydrolytic enzymes, receptors, adhesion molecules and complement regulatory proteins [12, 13]. The biosynthetic pathway is mediated by sequential additions of sugars and other components to phosphatidylinositol (Figure 1A) [12–14]. The cell surface expression of GPI-anchored proteins involves a total of 26 genes, which, with the exception of the X chromosome-linked phosphatidylinositol glycan anchor biosynthesis, class A (*Piga*) gene, are widely distributed throughout the autosomes (Table 1). *Clostridium septicum* α-toxin selectively binds GPI-anchored proteins at the cell surface and kills host cells [15]. A defect in the GPI-anchor biosynthetic pathway does not affect ESC proliferation *per se*[16], but a loss-of-function mutation in the pathway results in an α-toxin resistant phenotype, providing a platform for positive selection screening.

**Table 1:** **Genes involved in GPI-anchor biosynthesis and their mutant alleles**

			No. of mutant alleles		
Gene	Chr	CDS length (bp)	H129-2	HAP-1	Total
*Pigc*	1	891	2	2	4
*Pigm*	1	1269	5	4	9
*Pign*	1	2793	NA*	NA*	NA*
*Dpm1*	2	780	2†	3	5
*Dpm2*	2	252	0	1	1
*Pigt*	2	1746	4	4	8
*Pigu*	2	1305	10	3	13
*Dpm3*	3	276	0	1	1
*Pigk*	3	1185	7	1	8
*Pigo*	4	3303	5	4	9
*Pigv*	4	1479	5†	6	11
*Pigg*	5	2925	NA*	NA*	NA*
*Pgap2*	7	750	1	3	4
*Pigb*	9	1626	7	3	10
*Pigy*	9	213	0	0	0
*Mpdu1*	11	741	0	0	0
*Pigl*	11	756	3	1	4
*Pigs*	11	1665	3	0	3
*Pigw*	11	1509	1	1	2
*Pigh*	12	564	1	0	1
*Gpaa1*	15	1863	6	1	7
*Pigp*	16	396	1	1	2
*Pigx*	16	756	6	3	9
*Pigf*	17	657	2	2	4
*Pigq*	17	1743	NA*	NA*	NA*
*Piga*	X	1455	NA*	NA*	NA*
		(Total)	71	44	115

*Four genes excluded from screening targets (see text).

**Figure 2:**
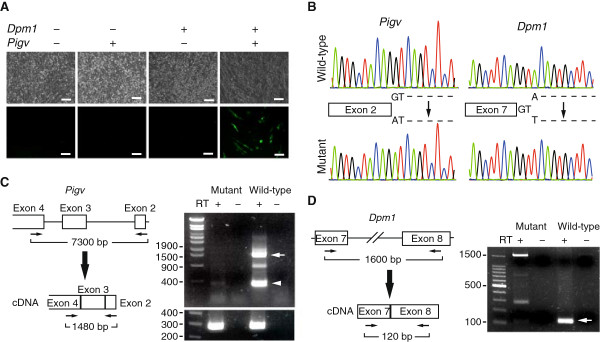
***Pigv*** **and** ***Dpm1*** **are simultaneously mutated in clone B502. (A)** Functional complementation assay of clone B502 ESCs. Co-transfection of both *Dpm1*- and *Pigv*-encoding plasmids was necessary for expression of the GPI-anchored GFP reporter at the cell surface. Original magnification, ×200. Scale bars, 100 μm. **(B)** Sequence chromatograms of wild-type (upper) and mutant (lower) ESCs at *Pigv* (left) and *Dpm1* (right) loci. Point mutations at the consensus 5′ splice site sequence (GT) of *Pigv* intron 2 (c.78 + 1G > A) and at the nucleotide next to the consensus 5′ splice site sequence (GT) of *Dpm1* intron 7 (c.563 + 3A > T) are shown. **(C)** Reverse transcription polymerase chain reaction (RT-PCR) of *Pigv* illustrating that intron 2 is correctly spliced in wild-type but not in mutant ESCs. The arrow indicates a transcript containing an enzymatically important region [21]; the arrowhead putatively indicates a short isoform. The left lane of the electrophoretic gels represents molecular size markers. Note that cDNA integrity of mutant ESCs was confirmed by amplification of *Actb* (lower gel). **(D)** RT-PCR of *Dpm1* illustrating that intron 7 is correctly spliced in wild-type (white arrow) but not in mutant ESCs. The left lane represents size markers.

†One H129-2 clone has two mutant alleles (see Table 2 and Figure 2).

### ENU mutagenesis

Unlike some viral or transposon-based insertional mutagens, the DNA alkylating agent ENU can introduce a high rate of point mutations into the genome, irrespective of whether it hits active genes, inactive genes, or intergenic regions [17, 18]. In the present study, we used two germline competent haploid mouse ESC lines, H129-2 and HAP-1 [7, 19]. After the enrichment of haploid cells by flow sorting (Additional file 1: Figure S1A and B), H129-2 and HAP-1 ESCs were treated with 0.25 and 0.20 mg/ml of ENU, respectively (Figure 1B). For the purpose of further increasing the mutation rate, pretreatment with the alkyltransferase inhibitor O^6^-benzylguanine (O^6^-BG, 10 μM) [20] was also included in the experimental design. Most cells were killed by these treatments [20], and the residual surviving cells (0.13–0.49%; *n* = 4) were treated with 1 nM α-toxin to select for GPI-anchor pathway mutants.

A total of 114 resistant clones (70 from H129-2 and 44 from HAP-1) were separately isolated and subjected to the previously described functional complementation assay [4] involving transfection with various combinations of plasmids encoding the 26 candidate genes (for details, see Methods section). As a reporter, we co-transfected ESCs with a plasmid encoding green fluorescent protein (GFP) fused with a GPI-anchor attachment motif (GFP-GPI; see Figure 1C). Only when the mutated gene(s) were exogenously restored did the cells express GFP at their surface, enabling mutants to be identified by their fluorescence. Representatively, four ESC clones were also analyzed by Sanger sequencing and their causative mutations were detected in the corresponding GPI-pathway genes (Additional file 2: Figure S2). Eventually, all isolated 114 clones harbored at least one mutant allele (115 mutant alleles in total) in any of 20 known genes involved in the GPI-anchor biosynthetic pathway (Table 1).

Among the 114 mutant ESC clones, clone B502 exhibited fluorescence when transfected with a mixture of cDNA-expressing vectors for all known GPI-anchor pathway genes (26 cDNAs in total), not when transfected with a single gene plasmid. A series of step-wise reductions in the repertoire of cDNA-expressing vectors revealed that both *Dpm1* and *Pigv* cDNAs were essential to restore the fluorescence (Figure 2A). Sanger sequencing of genomic DNA from the B502 ESC clone identified causative point mutations of both genes (Figure 2B–D). Thus, the appearance of this double mutant in our screening suggests that the mutagenicity of ENU is of a sufficiently high level for saturation mutagenesis.

Haploid ESCs have an inherent tendency to become diploid during culture [7–11]. This process, autodiploidization, resulted in undesirable but inevitable contamination of the diploidized ESCs (Additional file 1: Figure S1A). In this study, 86.0% of ESCs remained haploid at the point of ENU treatment (*n* = 3) and the rest were diploid. In the diploidized ESCs, because either of the duplicated X chromosomes could undergo X-inactivation or chromosomal loss, an ENU-induced mutation on the other allele of the X-linked gene would immediately lead to a complete loss-of-function. This meant that, in the mixture of haploid and diploid ESCs, X-linked mutants would be more frequently obtained than autosomal recessive mutants. In the present study, prior to mutagenesis with ENU, we introduced extra copies of human *PIGA* cDNA into the H129-2 haploid ESC line, but not into the HAP-1 haploid ESC line. As a result, X-linked *Piga* mutants apparently dominated in the HAP-1 ESC population (53.6% of mutant clones), whereas no *Piga* mutants, but instead various other autosomal mutants, appeared in the H129-2 ESC population.

*Pigg*, *Pign*, and *Pigq* are known by their hypomorphic loss-of-function phenotypes [13, 22, 23]. Consistently, a recent ESC-based mutagenesis study using the clustered regularly interspaced short palindromic repeats (CRISPR)-Cas system also screened for the resistance to α-toxin and failed to obtain *Pigg*, *Pign*, and *Pigq* mutants [24]. This suggests that, apart from these three genes and *Piga*, the remaining 22 genes are essential for the maintenance of α-toxin sensitivity in our screening scheme. Because the present mutagenesis study identified 20 of the 22 essential genes, this corresponded to a degree of saturation of 91%.

### Whole-exome sequencing for detection of mutations in haploid ESCs

The degree of saturation in mutagenesis largely depends on the mutagen. We examined 10 independent H129-2-derived mutant ESC clones by whole-exome sequencing (WES) and compared the results with those from ENU-untreated parental ESC clones (Table 2 and Additional file 3: Tables S1–S10). Over 98% of the reads were successfully mapped to the NCBI37/mm9 mouse reference genome with a mean coverage of 92.4, and 84.5% of the exome regions were analyzed at >30-fold depth (Additional file 4: Figure S3). Given that each locus has in principle one allele, only mutations designated “homozygous” were taken into account by our WES analytical pipeline (see Methods).

**Table 2:** **Mutations identified by whole-exome sequencing**

			(SnpEff§) High				Moderate	Low		Modifier
Clone name	Responsible genes*	Total no. of mutations	Nonsense mutations	Splice site alterations	Frameshift mutations	Loss of start codon	Nonsynonymous mutations ***etc.*** ||	Synonymous mutations	Others¶	
B3-1	*Pigt*	220	6	5	0	0	115	39	1	54
B5-1	*Dpm1*	297	6	3	0	1	128	60	2	97
B7-2	*Pigo*	218	5	1	0	0	103	38	1	70
B102	*Pigb*	10	0	0	1	0	4	0	0	5
B201	*Pigk*†	41	1	1	0	0	14	7	0	18
B502	*Dpm1, Pigv*	511	13	12	1	1	241	110	5	128
B1001	*Pigv*	306	8	2	0	0	127	55	1	113
B1002	*Pigs*	330	10	2	1	0	152	63	0	102
B1007	*Gpaa1*	23	1	1	0	0	9	2	1	9
F-43	*Pigo*‡	375	12	4	2	0	153	83	2	119
Average		233.1	10				104.6	47		71.5

*Genes whose mutations are responsible for the host cell phenotypes.

†All exons are deleted (Additional file 7: Figure S4).

‡Undetectable because of an exome design defect (Additional file 2: Figure S2).

§Also explained in Additional file 6: Table S12.

||The remainder: one codon insertion in clone B102.

¶Others: start gained; synonymous stop.

To filter out potential false-positive mutations, we adopted the following criteria: the mutations are only positive (i.e., “true-positive”) when >90% of reads are called “alteration” in ENU-treated mutant ESCs and >95% of the corresponding reads are called “reference” in ENU-untreated control ESCs. These criteria were validated by the Sanger sequencing of 20 “true-positive” mutations and 22 “false-positive” mutations in mutant clone F-43. As a result, 19 of the 20 “true-positive” mutations were confirmed, and 0 of the 22 “false-positive” mutations were detected. One mutation thought to be “true-positive” but not detected by Sanger sequencing was near the threshold line of our criteria. Using Fisher’s exact test, we obtained an extremely small *P* value (*P* = 4.5 × 10^−11^), confirming the appropriateness of the above criteria for filtering WES data. Yoshida *et al.* previously reported a true-positive rate of candidate mutations of only 53.9% using WES data from patients with myelodysplastic syndrome (MDS) [25], indicating that the haploid nature of our mutant cells is advantageous for the accurate determination of mutations compared with diploid cells such as those of MDS patients.

### Frequency and characteristics of ENU-induced mutations

We detected 10–511 mutations in the 49.3-Mb exome region of each clone (Table 2). The average number of mutations was 233.1, representing an overall mutation rate of 4.72 Mb^−1^. Although these 10 clones were mutagenized with ENU under the same conditions, the number of mutations diverged between clones for as yet unknown reasons. Nevertheless, the overall mutation rate is comparable to that of previous reports [18, 26, 27].

**Figure 3:**
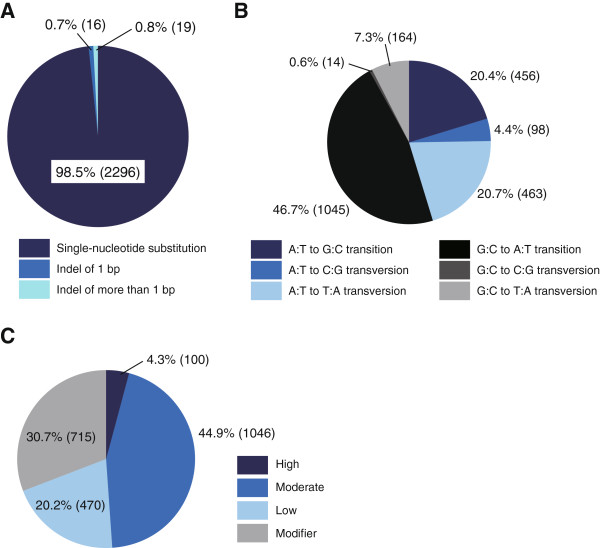
**Characteristics of ENU mutagenesis in haploid ESCs. (A)** Mutation types detected by whole-exome analysis. Actual numbers of mutations are shown in parentheses through Figure 3A–C. **(B)** Frequencies of nucleotide changes. The 2,240 point mutations are classified as transitions or transversions. Fewer than 2.5% of the 2,296 single-nucleotide substitutions could not be classified by our WES analytical pipeline for unknown reasons. We excluded these data from the analysis in Figure 3B. **(C)** Effects of mutations on genes predicted by SnpEff software (see also Additional file 6: Table S12). If more than one effect was annotated on a single mutation, effects with higher impact prediction were adopted.

Most (98.5%) mutations were single-nucleotide substitutions (Figure 3A) [3, 18, 28]. Among a total of 2,240 single-nucleotide substitutions, the A:T base pair mutations (45.5%) comprised similar proportions of transitions (A:T to G:C, 20.4%) and transversions (A:T to C:G or T:A, 25.1%), whereas the G:C base pair mutations (54.6%) comprised a higher proportion of transitions (G:C to A:T, 46.7%) than transversions (G:C to C:G or T:A, 7.9%) (Figure 3B and Additional file 5: Table S11). According to previous reports in mice including whole-genome sequencing data, ENU-induced mutations were markedly biased toward mutations in A:T base pairs (74.2–87%) [17, 27, 29, 30], which is considered to be a serious obstacle for random mutagenesis. However, we observed a much smaller base pair preference for A:T (45.5%) or G:C (54.6%) at mutated positions within the exome. This may in part be reflective of a lower A:T content in exonic DNA regions (49.7%) compared with the entire genome (58.2%). Alkylating agents such as ENU cause alkylation at the O^6^ position of guanine, leading to the G:C to A:T transition [31], while an intrinsic repair mechanism mediated by O^6^-alkylguanine-alkyltransferase plays a key role in repair of O^6^-alkylguanine adducts. In this study, we pretreated haploid ESCs with the alkyltransferase inhibitor O^6^-BG. As a result, G:C to A:T transitions were predominantly observed, which contributes to a much smaller base pair preference between A:T and G:C. Indeed, O^6^-BG was not used in the abovementioned reports [17, 27, 29, 30].

We used SnpEff software (v3.2) to predict the impact of the mutations [32, 33]. In brief, a “high” impact is that assumed to be disruptive to the protein; a “moderate” impact mutation is non-disruptive but might change the effectiveness of the protein; a “low” impact is unlikely to be accompanied by a change in the protein behavior; and “modifier” impact variants usually occur in introns or affect noncoding genes [34] (representative examples are shown in Additional file 6: Table S12). This software classified one-half of the mutations as either “high” impact (i.e., nonsense, splice site, frameshift, or loss-of-start-codon) or “moderate” impact (i.e., nonsynonymous) mutations, which narrowed down the list of candidate genes (Table 2 and Figure 3C). Thus, the analysis pipeline successfully confirmed responsible mutations in nine out of 10 mutant ESC clones tested: seven harbored a point mutation of GPI-anchor pathway genes, categorized as high (three clones) or moderate (four clones) impact; one clone was confirmed to harbor point mutations that affected two genes (*Dpm1* and *Pigv*) as described above (Figure 2); and one harbored a large deletion encompassing all exons of the *Pigk* gene (Additional file 7: Figure S4). The rest (clone F-43) harbored a mutation in the sixth exon of *Pigo*, which was undetectable because of an accidental omission in the ready-made exome capture design, so was instead confirmed by Sanger sequencing (Additional file 2: Figure S2). The high success rate in mutation identification is a prerequisite, though is not sufficient, for the detection of novel genes. Our data demonstrate the major contribution of exonic mutations to the phenotypes [3] and the effectiveness of combining WES with a haploid-based ENU mutagenesis approach.

### Mutation rate for each gene depends on the coding sequence length

**Figure 4:**
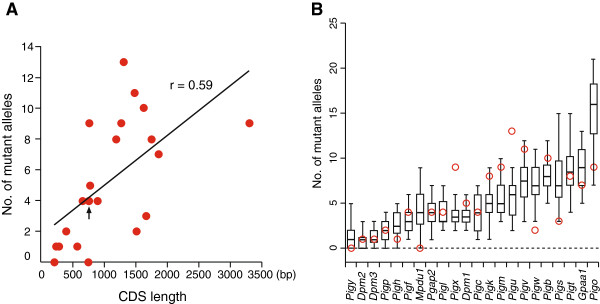
**ENU mutagenesis generates a CDS length-dependent rate of mutants. (A)** Scatter plot of 22-gene mutants according to CDS length and the number of independent mutant alleles (Pearson’s correlation r =0.59). An arrow indicates the overlap of the two plots (*Pgap2* and *Pigl*). **(B)** Box plots representing 20 runs of simulation for the number of mutant alleles. In this simulation, 115 mutagenic hits were generated and assigned to any of the 22 genes. Bars, boxes, and whiskers indicate medians, upper and lower quartiles, and the lowest and highest extremes still within 1.5 times the box sizes, respectively. Columns for the 22 genes are ordered according to CDS length. Experimental data from our functional complementation assay are overplotted (red circles). Notably, 20 of the 22 genes are mutated in at least one ESC clone.

The efficient and unbiased nature of ENU as a mutagen, combined with the haploid ESC system, enabled a realistic experimental design of mammalian saturation mutagenesis screening to be performed in a resource-saving manner. As described above, we isolated 115 independent mutant alleles whose causative mutations covered 20 out of 22 GPI-anchor pathway essential genes. Since most ENU-induced mutations were single-nucleotide substitutions (Figure 3A and B), we speculated that the mutation rate for each gene depended on its coding sequence (CDS) length. To confirm this, we plotted the number of mutant alleles for each responsible gene against its CDS length (Figure 4A), revealing a positive proportional correlation (Pearson’s correlation r = 0.59). This result was further validated by comparison with Monte Carlo simulation: 20 runs of 115 mutagenic hits were assigned to any of the 22 genes essential for the pathway with a probability proportional to their CDS length (Figure 4B). Besides a few outliers, the experimental mutation rate for each gene was within the range of the simulation predictions, indicating the relevance of the CDS length as a prime determinant of gene mutation rates in this system.

### Modeling the experimental time course of the appearance of mutant alleles

**Figure 5:**
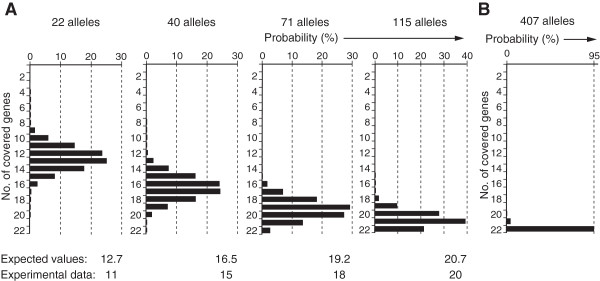
**Number of essential genes covered by 22, 40, 71, and 115 mutant alleles. (A)** The number of essential genes covered by 22, 40, 71, and 115 mutant alleles for the GPI-anchor pathway. Histograms represent the simulative probability distribution of the covered gene numbers using CDS length-dependent mutation rates. Expected values and experimental data are compared at the bottom of each panel. Notably, the expected values based on the CDS length-dependent mutation rates are more consistent with the experimental data than those based on the identical mutation rates (see Additional file 8: Figure S5). **(B)** The simulated number of genes covered by 407 mutant alleles.

We next attempted to trace the experimental time course of the appearance of mutant alleles by simulation. During the experiment, the number of mutant alleles obtained from α-toxin selection accumulated in a step-wise manner: 22, 40, 71, and 115 mutant alleles, which covered 11, 15, 18, and 20 of the essential genes, respectively. The experimentally-determined gene number at each step was compared with the expected gene number obtained by simulation. Under the simplest assumption that mutation rates are identical for each gene, the expected gene number did not match the corresponding experimental result at each step (Additional file 8: Figure S5). By contrast, under the current assumption that mutation rates vary proportionally to CDS length, the expected gene numbers better fitted the experimental results (Figure 5A). When the recovered allele number is accumulated up to 407, the expected gene number will reach 22, saturating all the known genes essential for this pathway (Figure 5B). In other words, to achieve complete saturation, isolation of around 400 mutant clones will be required in this experimental setting.

### Extended model for estimation of gene number in a biological pathway

**Figure 6:**
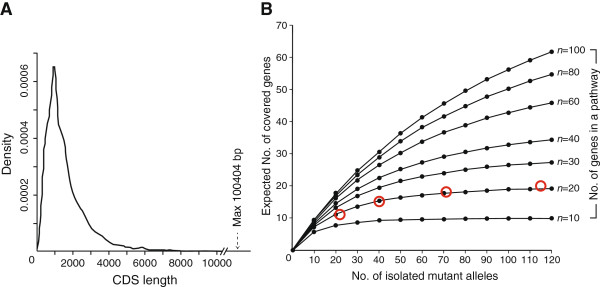
**Experiment-based prediction of essential gene number for a biological process. (A)** Kernel density estimation representing the distribution of CDS length for all mouse genes. **(B)** Regression curves of simulation results. Plotting of experimental data, e.g., our present data (red circles), allows the prediction of the number of genes essential for a given pathway.

Finally, we extended this experimental and simulative study of ENU mutagenesis to a general biological process. Two critical prerequisites were a mutagenic scheme of random single-nucleotide substitutions and independent isolation of different mutant alleles. The former contributes to the assumption of the CDS length-dependent gene mutation rates and the latter contributes to an accurate enumeration of the screened mutants. Assuming that a variable number (*n*) of genes is essential for a biological pathway, the CDS length for each gene can be assigned according to the distribution of CDS length for all mouse genes deduced from the consensus coding sequence (CCDS) database (Figure 6A) [35]. Using the putative CDS length as a parameter for the simulation, we plotted the expected numbers of covered genes against the cumulative numbers of isolated mutant alleles, where the essential gene number, *n*, varies from 10 to 100 in increments of 10 (Figure 6B).

Importantly, our experimental data (i.e., the step-wise increase of identified gene number at each step of allele collection) demonstrated a close fit to the simulated curve for *n* = 20, which provides an accurate estimate of the number of GPI-anchor pathway genes. Using the simulation curves, even a smaller-scale pilot experiment that screens as few as 30–50 mutant clones can provide an estimate of the essential gene number for a given biological process. This could serve as an advantage of ENU mutagenesis because the CDS length-dependent mutation rate is not applicable to other widely-used mutageneses such as gene-trap [36–38].

## Discussion

In this study, we examined the feasibility of “saturation” mutagenesis and screening for recessive traits in mammals by exploiting haploid mouse ESCs where single-allele loss-of-function mutations could immediately manifest their phenotypes. To obtain a high mutation rate, we used the classical alkylating chemical mutagen ENU in conjunction with pretreatment with the alkyltransferase inhibitor O^6^-BG. Mutations were screened by a plasmid-based functional complementation assay and were further assessed by WES. As a result, most exome mutations were single-nucleotide substitutions with little base pair preference, guaranteeing the randomness of mutagenesis in this approach. The haploid nature of the genome provided a significant advantage for reliable single-nucleotide variant (SNV) calling, compared with heterozygous SNV detection in the diploid genomes. Thus, the combination of the haploid ESC system and WES, as two modern technologies, sheds a new light on the classical mutagenesis approach with ENU.

An important contribution of this study is that we demonstrated the CDS length-dependent allele frequency of each gene when mutated with ENU. Introducing the CDS lengths as a parameter was a good way to simulate the GPI-anchor pathway-mutant screening that was based on the functional complementation assay using candidate gene-expression plasmids. This simulation principle could also be extrapolated to similar mutagenesis experiments for other biological pathways. Most biological pathways have been comprehensively investigated and at least some of their genetic components are already known [39]. Therefore, it is important to understand how many other relevant genes remain to be identified and when the screening would reach saturation. For this purpose, by taking advantage of the simulation results in Figure 6B, the ENU mutagenesis of haploid ESCs and a subsequent small-scale pilot screening experiment would provide a good estimate of the number of essential genes in a pathway of interest.

The WES analysis could also contribute to the construction of a list of candidate genes. In a recent review [40], Schneeberger notes that next-generation sequencing-based methods for mutation identification will soon replace other methods such as genetic mapping in forward genetic screens. The power of WES in detecting ENU-induced mutations is also discussed, and the analysis of pooled genomes is recommended to manage large numbers of samples. Indeed, considering the much higher accuracy of mutation identification achieved in haploid compared with diploid cells, deep-sequencing of pooled DNA samples from multiple isolated clones may be a cost-effective way to identify mutations. Typically it appears that when accumulating sequenced clones in a positive-selection screening approach, mutations will be enriched in a limited number of genes. Further, the more often different mutations are found within a gene, the more likely the gene will be a target of the screening. Notably, the mutant allele frequency should be normalized by dividing it by the CDS length, such that a gene with a high mutant allele frequency and smaller CDS should be given priority over that with a high allele frequency and larger CDS.

In addition to the GPI-anchor biosynthetic pathway we used here, our screening strategy is applicable to many other experimental systems in which positive selection of mutant clones is available. At present, several biological pathways can be proposed as plausible targets for forward genetic screens, including mismatch repair genes (selected by 6-thioguanine) [7, 24], genes involved in the exit from the pluripotency circuit of ES cells (selected by the Rex1–GFP reporter) [37], genes involved in tumor necrosis factor (TNF) receptor 1 signaling (selected by TNF-α) [36], and effector genes of extrinsic apoptotic stimuli (selected by TNF-related apoptosis-inducing ligand) [36]. Furthermore, our strategy could be useful for investigating the pathways related to drugs and toxins with undetermined or partially determined mechanisms. For example, targets of the poly (ADP–ribose) polymerase 1/2 inhibitor olaparib [41] and effector genes of ricin toxicity [8] are currently under investigation. Many more compounds not yet examined could also be considered candidates of our strategy.

The GPI-anchor biosynthetic pathway has been extensively characterized [12–14], and the probability of new gene discovery is limited. In the present screening, 20 out of the 22 known essential genes were identified. Identification of a 23rd gene will depend on its CDS length. If this is similar to the average CDS length of the other 22 known genes (approx. 1,090 bp), the 23rd gene should appear among the isolated 115 alleles with a probability of >99% (see Methods). If its CDS length is shorter (e.g., 213 bp, like *Pigy*), its discovery probability is 64%. Therefore, the 23rd gene, if it exists, is likely to have a shorter than average CDS length otherwise its existence is less likely. Indeed, the recent screening study using the lentiviral CRISPR-guide RNA library identified no additional GPI-anchor pathway genes when the same concentration of α-toxin (1.0 nM) was used for positive selection [24]. However, when a lower toxin concentration (0.50 nM) was used, at least four additional genes, *B4galt7*, *1700016K19Rik*, *Cstf3*, and *Ext*2, were identified as modulators of α-toxin susceptibility, although they do not directly affect the GPI-anchor biosynthetic pathway. Taken together, these results suggest that mutagenesis screening of the GPI-anchor biosynthetic pathway has almost been saturated with the 22 known essential genes.

The mutation numbers showed a marked variation and non-normal distribution (Table 2) although the 10 clones were mutagenized with ENU under the same conditions. Thus far, we do not fully understand this phenomenon because, to our knowledge, there are no other reports of haploid cells being ENU-mutagenized and their genome deep-sequenced. It could reflect the relatively small number of mutant clones examined in this study, but further investigation is necessary to clarify this because a skewed distribution of ENU susceptibility may be a limitation of our method, which is based on the randomness of ENU mutagenesis.

## Conclusions

In summary, ENU mutagenesis of the haploid mouse ESC system allowed for a high level of saturation mutagenesis and recessive loss-of-function screening that would have been difficult to achieve in mammalian cells. WES analysis revealed that although ENU randomly created point mutations in both genic and intergenic regions, nevertheless, most of the phenotype-causing mutations were clustered in exons, providing a rationale for the application of WES technology. Importantly, ENU-induced mutations affected each gene at a rate proportional to its CDS length. This contributed to a reliable estimation of the number of genes essential for a pathway under investigation, by way of computational simulation based on a small-scale pilot experiment. This study opens up new opportunities for drug or toxin target screening.

## Methods

### Maintenance and purification of haploid ESCs

H129-2 and HAP-1 haploid ESC lines were kind gifts from M. Leeb [7]. They were maintained in chemically defined 2i medium [7, 19] consisting of a 1:1 mixture of Dulbecco’s modified Eagle’s medium/F12 (DMEM/F12) and neurobasal medium, supplemented with N-2, B-27, non-essential amino acids, L-glutamine, 0.35% bovine serum albumin fraction V (all from Life Technologies), 0.1 mM 2-mercaptoethanol (Sigma-Aldrich), 1,000 U/ml of mouse leukemia inhibitory factor (mLIF; Millipore), and 2i (1 μM of PD0325901 and 3 μM of CHIR99021; both from Axon). To purify the haploid cell fraction, ESCs were stained with 15 μg/ml of Hoechst 33342 (Life Technologies) at 37°C for 30 min, then the cells on 1n peak were sorted with the BD FACSAria II Cell-Sorting System (BD Biosciences). Sorted cells were plated onto a 24-well plate or a T-25 flask with or without mitomycin C-treated mouse embryonic fibroblast (MEF) feeders, and were used for ENU mutagenesis after several days. DNA contents of the ESCs were evaluated using BD FACSCanto II (BD Biosciences) after fixation in ethanol, RNase digestion, and staining with propidium iodide (Sigma-Aldrich). Data analyses were performed using BD FACSDiva software (BD Biosciences) or FlowJo software (TreeStar).

### Mutagenesis protocols

ENU was purchased from Sigma-Aldrich and was dissolved and diluted as previously described [20]. As a pretreatment, O^6^-BG (10 μM; Sigma-Aldrich), an inhibitor of O^6^-alkylguanine-alkyltransferase [20], was added to the culture medium from 1 day before to 1 day after the ENU treatment. Random mutations were introduced into haploid ESCs by treating them with ENU (0.25 mg/ml for H129-2; 0.2 mg/ml for HAP-1) at 37°C for 2 h. After the mutagenesis step, cells were cultured on MEF feeders in Knockout DMEM supplemented with 20% fetal bovine serum (both from Life Technologies), non-essential amino acids, L-glutamine, 2-mercaptoethanol, and mLIF. One week later, ESCs were plated on 0.1% gelatin-coated tissue culture plates and treated with 1 nM of α-toxin at 37°C for 24 h. After removal of α-toxin, plates were incubated for 6 or 7 days until individual colonies appeared. Colonies were separately isolated and subjected to functional complementation assays.

### Functional complementation assay

Independently-isolated mutant ESC clones were transfected with expression plasmids of candidate genes, together with the GFP-GPI reporter plasmid, using TransFast Transfection Reagent (Promega) according to the manufacturer’s instructions. First, a mixture of cDNA-expressing plasmids for all known GPI-anchor pathway genes (26 cDNAs in total) was transfected into each mutant clone. One or 2 days later, GFP fluorescence was observed using an IX70 inverted microscope (Olympus) or BioRevo BZ-9000 inverted microscope (Keyence). Next, the repertoire of the cDNA-expressing plasmids in the mixture was reduced in a step-wise manner to narrow down the list of candidate genes. Finally, each mutant ESC clone was examined to determine whether a single gene plasmid could restore the mutant phenotype.

### Karyotype analysis

Karyotype analysis was performed as previously described [6]. Images were taken using the Olympus IX70 inverted microscope with the CoolSNAP cf CCD camera (Photometrics).

### Reverse transcription polymerase chain reaction of *Pigv*and *Dpm1*

Total RNA was isolated using the RNeasy Plus Micro Kit (Qiagen) and converted to cDNA by the SuperScript III First Strand Synthesis System (Life Technologies). Reverse transcription polymerase chain reaction (RT-PCR) was performed using KOD FX (Toyobo) with the following primers: *Pigv*, 5′-ATT TAG AAG CCG GAG GAA GCT CAG TC-3′ (forward) and 5′-CCA GTA GGT CAG GAA GTA GAC CAG AAC-3′ (reverse); *Dpm1*, 5′-CTC AAA TTC TGC TGA GAC CTG GAG CGT CAG-3′ (forward) and 5′-CCA TCT GAA AGA CAT AGC CTT TGG AGA CAC-3′ (reverse); *Actb*, 5′-CAG GGT GTG ATG GTG GGA ATG GGT CAG AAG-3′ (forward) and 5′-TAC GTA CAT GGC TGG GGT GTT GAA GGT CTC-3′ (reverse).

### Exome-enriched library preparation and exome sequencing

Genomic DNAs were extracted from 10 mutant ESC clones and two ENU-untreated parental ESCs using the DNeasy blood & tissue kit (Qiagen), and subjected to quality assessment with gel electrophoresis and measurement of DNA concentration. Exome-enriched libraries were generated using the Agilent SureSelect^XT^ mouse all exon kit following the manufacturer’s recommendation. In brief, genomic DNAs were sheared by sonication to generate fragments a few hundred base pairs in length, followed by end repair, A-tailing and adaptor-ligation of both ends. The resulting DNA fragments were then pulled down by hybridization with the SureSelect oligo capture library, and were PCR amplified using primers with indexes to generate sequence libraries. These libraries were subjected to paired-end (75–100 bp) sequencing on an Illumina HiSeq 2000 sequencer.

### Exome sequencing data processing

Raw data from Illumina sequencers were processed using the exome analysis pipeline (Amelieff). The pipeline performed the following steps. Some sequence data were provided in BAM format and were converted to fastq format with bam2fastx (tophat-2.0.4) [42] for use in the pipeline. First, raw sequence data were cleaned up with QCleaner software (Amelieff): low-quality reads (>20% of the base calls with a Phred score <20) were discarded; reads shorter than 32 and reads containing five or more N were also discarded; base calls at both ends with a Phred score <20 were trimmed. Quality checks on the raw and cleaned-up sequence data were performed by FastQC (v0.10.0) [43]. Next, the cleaned-up sequence data in fastq format were aligned to the NCBI37/mm9 mouse reference genome, which was fused with the decoy sequence to map the exogenous expression vectors for *PIGA*. BWA (v0.6.1) [44] with default settings was used to generate mapped data files written in SAM format. These were then converted to BAM format with SAMtools (v0.1.18) [45], followed by sorting. Duplicate reads were removed by Picard-tools (v1.75) [46]. Before and after the removal of duplicate reads, BAM files were indexed and coverage was calibrated. BED files of the exome-enriched regions were provided by Agilent [47].

After the elimination of duplicate reads, candidate regions were extracted and local realignment was performed with the Genome Analysis Toolkit (GATK v1.6-13) [48]. Recalibration of the base quality scores was performed with the same software. The resulting BAM files were visualized using the Integrative Genomics Viewer (IGV v2.1.21) [49]. SNVs and small indels were called by GATK’s UnifiedGenotyper using default settings, except for the modification of an optional parameter minIndelFrac from 0.25 to 0.2. SNVs and indels were filtered for sequencing and mapping qualities using the GATK VariantFiltration tool: in brief, SNVs and/or indels with low mapping quality, low sequence quality, low coverage, strand bias and adjacent homopolymers/SNVs were flagged. The average numbers of filtered SNVs and indels were 60,254 and 3,888, respectively, probably reflecting the genetic divergence between strains of the H129 haploid cell line and the C57BL/6 J mouse reference genome.

Finally, SNVs and small indels were annotated using SnpEff v3.2 software. The effects of mutations were categorized into four impact groups: high, moderate, low, and modifier [34]. Results were outputted in VCF format.

### Detection and validation of mutations

ENU-induced mutations were detected by comparing the VCF file of each mutant ESC clone with that of the ENU-untreated parental ESC clone using QmergeVCF software (Amelieff). As mentioned in the Results section, the mutations designated “homozygous” were filtered by the following criteria: when >90% of reads represented “alteration” in ENU-treated mutant ESCs and when >95% of reads represented “reference” in ENU-untreated control ESCs. Some mutations were validated by Sanger sequencing on an ABI Prism 3100 genetic analyzer (Life Technologies). Primers for amplification and sequencing are listed in Additional file 9: Table S13.

### Monte Carlo simulation of the number of GPI-anchor pathway genes identified by screening

Monte Carlo simulation was conducted under the assumption that the ENU-induced mutation rates vary proportionally to CDS length. CDSs of the 22 GPI-anchor pathway genes were extracted from the NCBI RefSeq database (release 56, November 2012). Conceptually, the 22 CDSs were concatenated into continuous blocks, of a total length of 23,982 nucleotides. Pseudorandom numbers were generated to designate the nucleotide coordinate along the concatenated blocks, and the coordinates were assigned back to the corresponding gene names. The operation was repeated as many times as the total number of mutant alleles (e.g., 115 times for 115 mutant alleles).

First, after repeating this operation 115 times, we counted the frequency of assignment to each gene. We ran this computer experiment consecutively 20 times and presented the results as box plots (Figure 4B). Next, to simulate the number of genes covered by 22, 40, 71, 115, and 407 mutant alleles, the assigned gene number for each screening step (i.e., 22, 40, 71, 115, and 407 times of operations) was counted. We generated 1 × 10^7^ simulated data sets for each step and created histograms by plotting the number of covered genes on the vertical axis and its probability on the horizontal axis. Expected values for the number of covered genes were calculated and compared with our experimental data. We also performed the simulation by assigning the equal CDS length to each gene under the simplest assumption that mutation rates are identical among the 22 genes. The simulation program was composed using Java SE development kit 7u25 (Oracle; the source code is provided in Additional file 10).

### Extended simulation of the number of genes essential for a biological pathway

For a given biological pathway containing a varying number (*n*) of genes, mutation rates for genes were assigned according to the distribution of CDS length in all mouse genes. The CDS lengths of all mouse genes were extracted from the CCDS database [50]. The extracted data of CDS lengths for 5, 15, 25, 35, 45, 55, 65, 75, 85, and 95 percentiles were 378, 615, 825, 954, 1,131, 1,365, 1,617, 2,022, 2,628, and 4,131 bp, respectively, representing the diversity of CDS lengths of all mouse genes. Conceptually, 10 bins were defined by these 10 representative CDS lengths (bp) and concatenated into continuous blocks. For example, for the simulation of a pathway consisting of 40 genes (*n* = 40), 4 × these 10 bins were concatenated to generate continuous blocks with a total length of 40 genes. The algorithms using pseudorandom numbers are described above. Expected numbers of covered genes (*y*-axis) were plotted against the number of mutant alleles (*x*-axis) for the varying number (*n*) of genes essential for a biological pathway (Figure 6B).

### Assessment of the probability of identifying an unknown gene

Providing that another unknown gene, in this case the 23rd gene, exists and has an average-sized CDS, the probability to miss this gene in each mutant allele is theoretically 22/23. Then, among the already isolated 115 alleles, the probability of its existence is calculated as follows:


### Supporting data

WES data from this project have been deposited [EMBL: ERP001518, DDBJ: DRP001798]. The other data sets supporting our results are included within this article and its additional files.

## Electronic supplementary material

Additional file 1: Figure S1: Haploid ESCs used in our experiments. **(A)** The DNA content of H129-2 ESCs was examined by propidium iodide staining. The left gate denotes haploid cells in S phase; the right gate diploid cells in S phase. The ratio between these two gates indicates that 89.0% of the cells are haploid. **(B)** Karyotype analysis of H129-2 ESCs. Twenty chromosomes are observed in each nucleus (dashed ovoid; original magnification, ×400). **(C)** The raw images of Figure 1C (original magnification, ×200). Note that ESCs were slightly damaged by cationic liposome-mediated gene transfer with TransFast. (PDF 4 MB)

Additional file 2: Figure S2: Sanger sequencing of mutations introduced by ENU. Nonsense mutations identified in exon 6 of *Pigo* (upper left), exon 10 of *Pigs* (upper middle), and exon 7 of *Pigu* (upper right). The frameshift mutation in exon 4 of *Pigx* is also shown (lower). (PDF 335 KB)

Additional file 3: Tables S1–S10: Detailed lists of mutations identified by WES. (XLSX 532 KB)

Additional file 4: Figure S3: Coverage of exome sequencing in H129-2 ESC clones. Shares of the regions with indicated depths are plotted for 12 (two, control; 10, mutant) H129-2 ESC clones. Controls 1 and 2 differ in insertion sites of the extra copies of *PIGA* cDNA. (PPTX 50 KB)

Additional file 5: Table S11: Characteristics of ENU mutagenesis in H129-2 ESCs. A total of 2,240 point mutations were classified by the modified base pairs and the types of DNA substitution (see also Figure 3B). (XLSX 47 KB)

Additional file 6: Table S12: Representative mutation effects annotated by SnpEff software. A complete description is provided in [34]. (XLSX 40 KB)

Additional file 7: Figure S4: Deletion of the *Pigk* locus in clone B201. **(A)** The coverage of mapped reads on *Pigk* exons is minimal (one or two) in clone B201, which contrasts with the multiple coverage on the same exons in control cells. Illumina sequencing data were visualized using IGV v2.1.21. **(B)** The coverage of mapped reads on the neighboring *Ak5* exons, 46 kb centromeric to *Pigk*, is recovered in clone B201 to a comparable coverage in control cells. **(C)** The coverage of mapped reads on the neighboring *St6galnac5* exons, 32 kb telomeric to *Pigk*, is recovered in clone B201 to a comparable coverage in control cells. (PDF 507 KB)

Additional file 8: Figure S5: Simulation of the covered gene numbers based on an identical mutation rate. The numbers of essential genes covered by 22, 40, 71, and 115 mutant alleles were simulated using an identical mutation rate for each gene. Histograms represent the simulative probability distribution of the covered gene numbers. Expected values and experimental data are compared at the bottom of each panel. (PDF 259 KB)

Additional file 9: Table S13: Primers used for validation of WES data. (XLSX 44 KB)

Additional file 10: **Source code used in our simulation.** (TXT 10 KB)

## Abbreviations

CCDS: Consensus coding sequence
CDS: Coding sequence
CRISPR: Clustered regularly interspaced short palindromic repeats
DMEM: Dulbecco’s modified Eagle’s medium
ENU: N-ethyl-N-nitrosourea
ER: Endoplasmic reticulum
ESC: Embryonic stem cell
GATK: Genome Analysis Toolkit
GFP: Green fluorescent protein
GPI-anchor: Glycosylphosphatidylinositol-anchor
IGV: Integrative Genomics Viewer
MDS: Myelodysplastic syndrome
MEF: Mouse embryonic fibroblast
mLIF: Mouse leukemia inhibitory factor
O^6^-BG: O^6^-benzylguanine
RT-PCR: Reverse transcription polymerase chain reaction
SNV: Single-nucleotide variant
TNF: Tumor necrosis factor
WES: Whole-exome sequencing.
