# Supplementary figures and images for: Simulation and estimation of gene number in a biological pathway using almost complete saturation mutagenesis screening of haploid mouse cells
Source: BMC Genomics. 2014 Nov 24;15(1):1016. doi: 10.1186/1471-2164-15-1016 (PMC4301880; doi:10.1186/1471-2164-15-1016)

**A**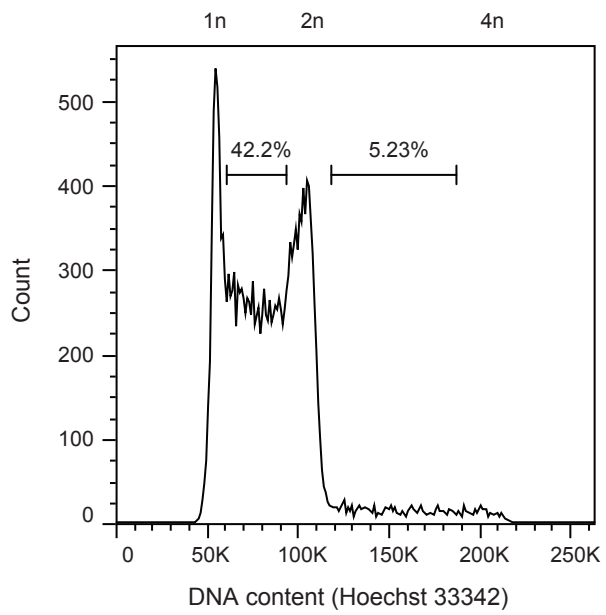**B**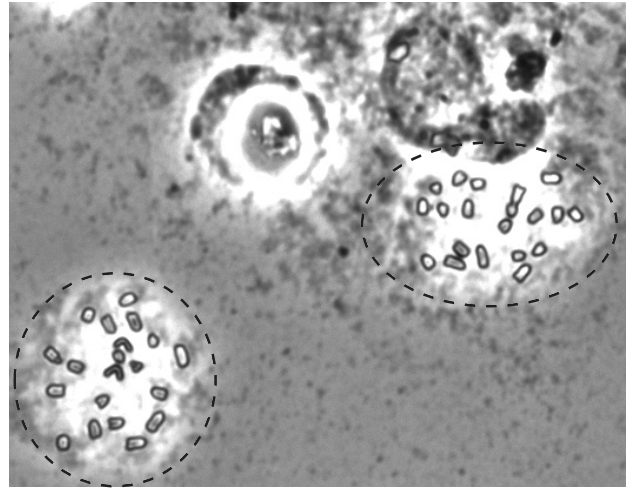**C**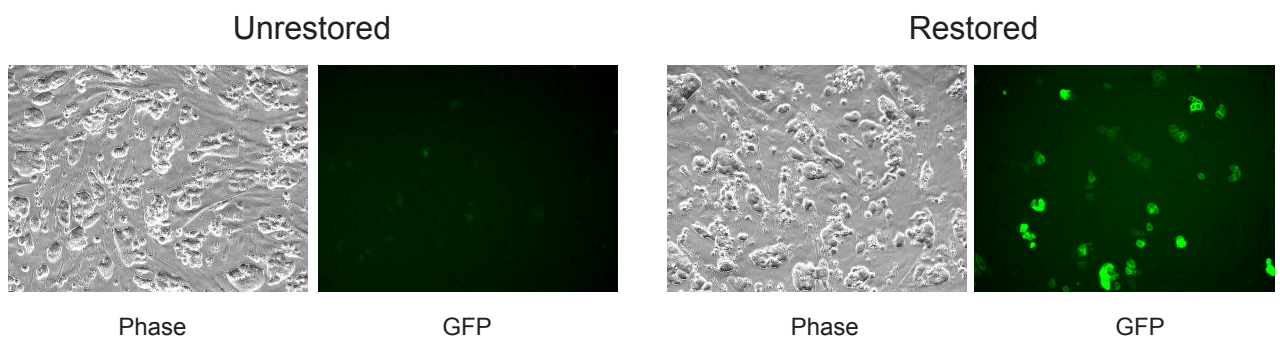

Supplement: Supplementary file 1 — Additional file 1: Figure S1: Haploid ESCs used in our experiments. (A) The DNA content of H129-2 ESCs was examined by propidium iodide staining. The left gate denotes haploid cells in S phase; the right gate diploid cells in S phase. The ratio between these two gates indicates that 89.0% of the cells are haploid. (B) Karyotype analysis of H129-2 ESCs. Twenty chromosomes are observed in each nucleus (dashed ovoid; original magnification, ×400). (C) The raw images of Figure 1C (original magnification, ×200). Note that ESCs were slightly damaged by cationic liposome-mediated gene transfer with TransFast. (PDF 4 MB) [file 12864_2014_6837_MOESM1_ESM.pdf]

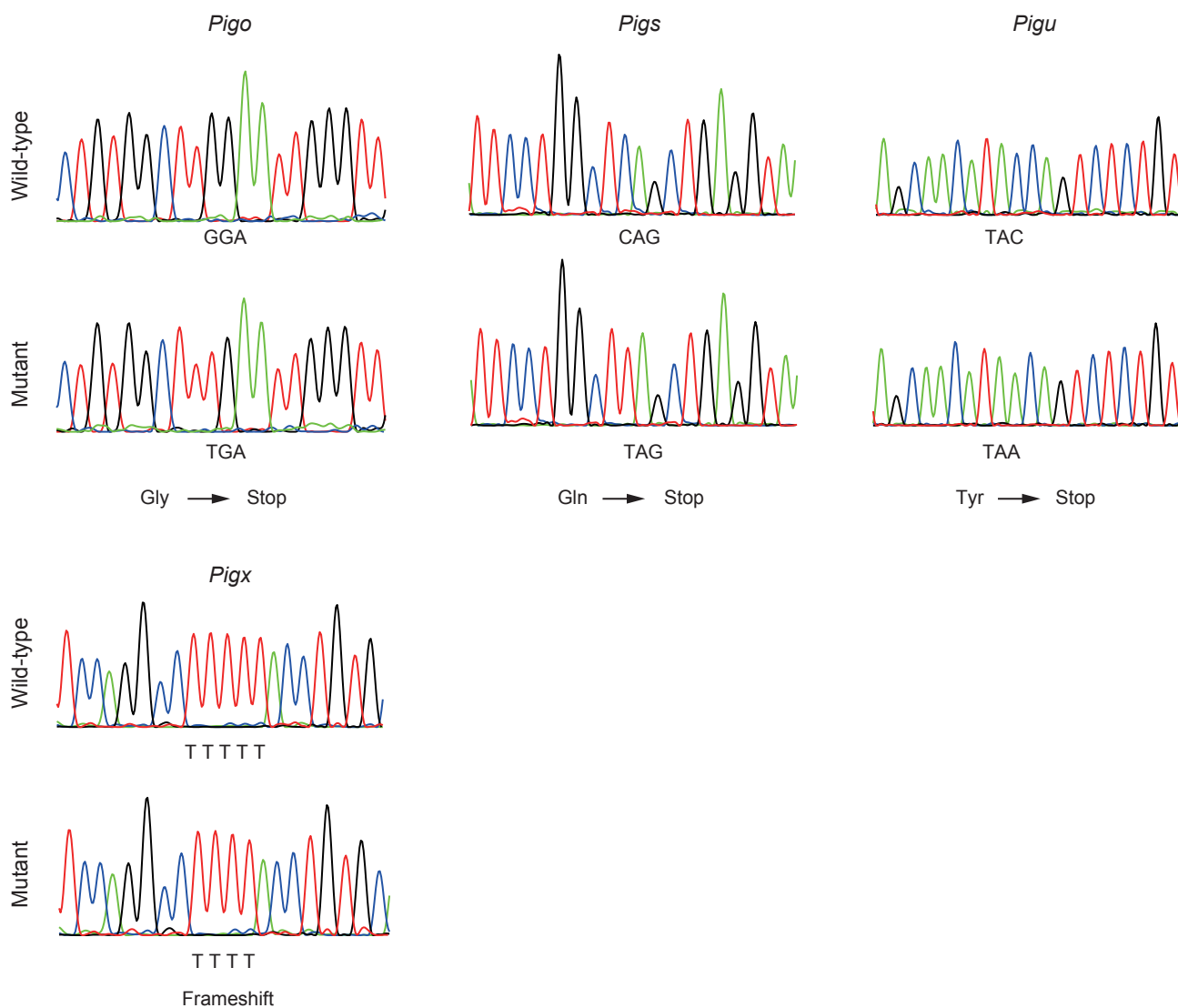

Supplement: Supplementary file 2 — Additional file 2: Figure S2: Sanger sequencing of mutations introduced by ENU. Nonsense mutations identified in exon 6 of Pigo (upper left), exon 10 of Pigs (upper middle), and exon 7 of Pigu (upper right). The frameshift mutation in exon 4 of Pigx is also shown (lower). (PDF 335 KB) [file 12864_2014_6837_MOESM2_ESM.pdf]

## Slide 1
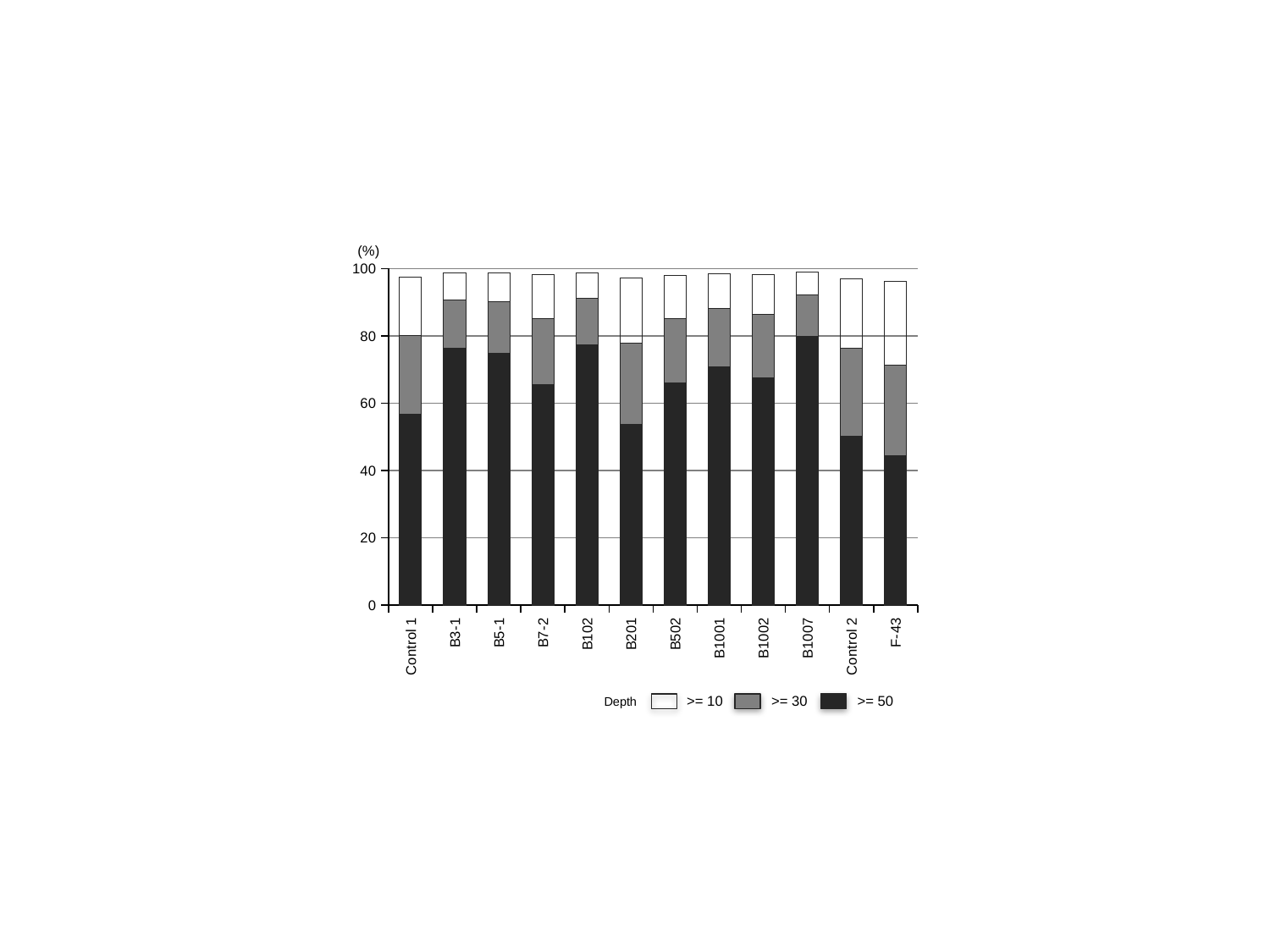

(%)
### Chart
| Category | | | |
|---|---|---|---|
| Control 1 | 56.6 | 23.4 | 17.5 |
| B3-1 | 76.3 | 14.3 | 8.100000000000009 |
| B5-1 | 74.8 | 15.40000000000001 | 8.6 |
| B7-2 | 65.6 | 19.5 | 13.10000000000001 |
| B102 | 77.2 | 13.9 | 7.700000000000003 |
| B201 | 53.6 | 24.3 | 19.3 |
| B502 | 66.0 | 19.2 | 12.8 |
| B1001 | 70.9 | 17.3 | 10.3 |
| B1002 | 67.5 | 18.8 | 12.0 |
| B1007 | 79.8 | 12.3 | 6.900000000000005 |
| Control 2 | 50.2 | 26.09999999999999 | 20.7 |
| F-43 | 44.3 | 27.10000000000001 | 24.69999999999999 |>= 10
>= 30
>= 50
Depth

Supplement: Supplementary file 4 — Additional file 4: Figure S3: Coverage of exome sequencing in H129-2 ESC clones. Shares of the regions with indicated depths are plotted for 12 (two, control; 10, mutant) H129-2 ESC clones. Controls 1 and 2 differ in insertion sites of the extra copies of PIGA cDNA. (PPTX 50 KB) [file 12864_2014_6837_MOESM4_ESM.pptx]

A

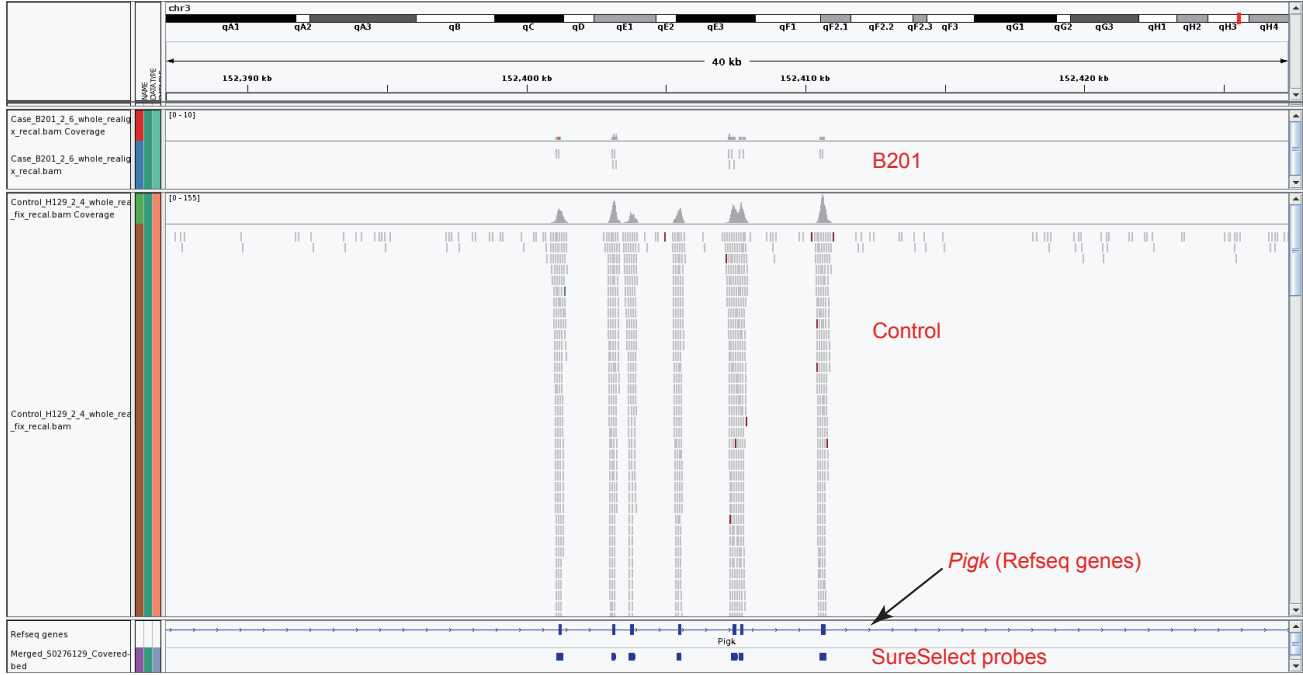

B

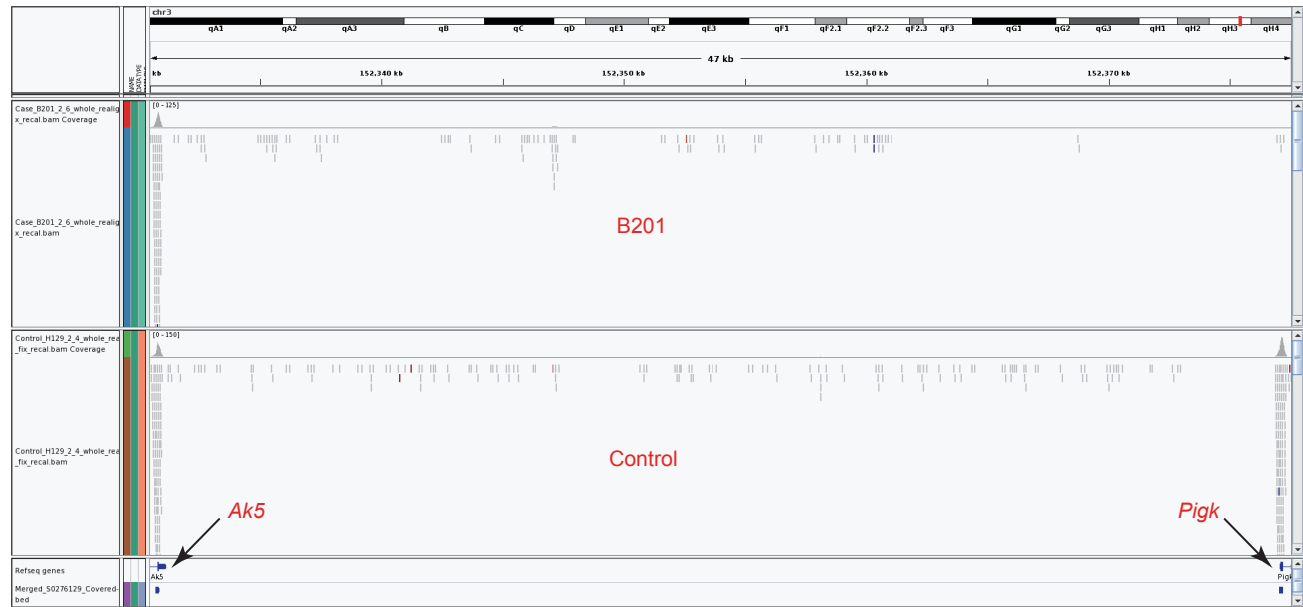

C

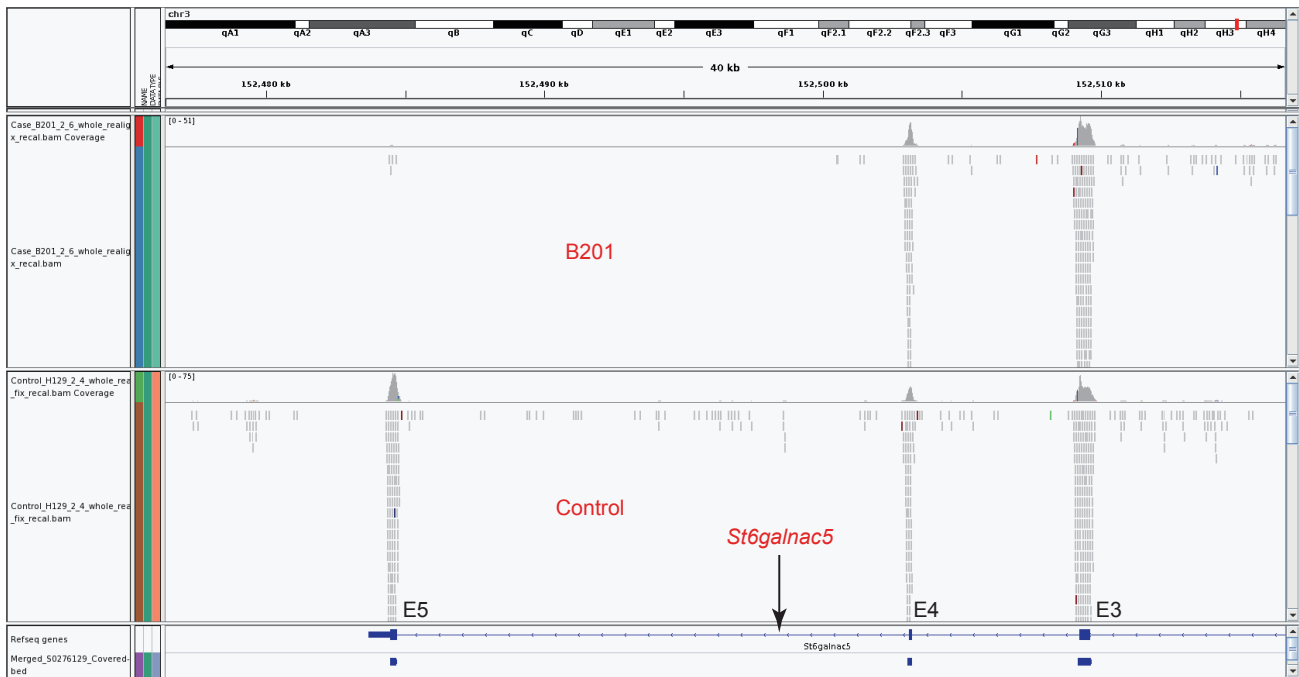

Supplement: Supplementary file 5 — Additional file 5: Table S11: Characteristics of ENU mutagenesis in H129-2 ESCs. A total of 2,240 point mutations were classified by the modified base pairs and the types of DNA substitution (see also Figure 3B). (XLSX 47 KB) [file 12864_2014_6837_MOESM5_ESM.pdf]

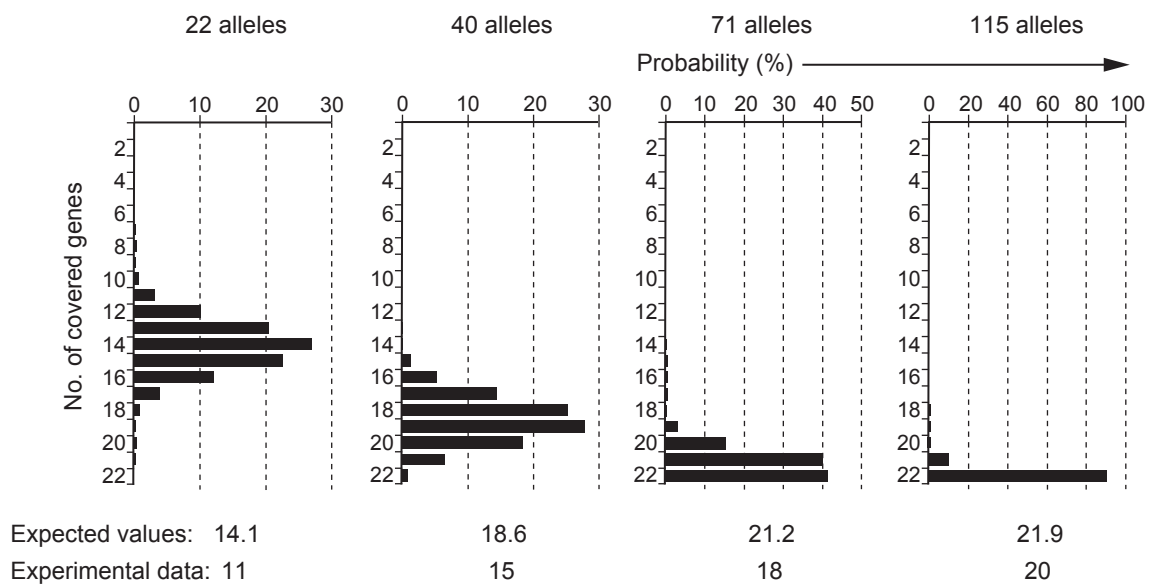

Supplement: Supplementary file 8 — Additional file 8: Figure S5: Simulation of the covered gene numbers based on an identical mutation rate. The numbers of essential genes covered by 22, 40, 71, and 115 mutant alleles were simulated using an identical mutation rate for each gene. Histograms represent the simulative probability distribution of the covered gene numbers. Expected values and experimental data are compared at the bottom of each panel. (PDF 259 KB) [file 12864_2014_6837_MOESM8_ESM.pdf]
